# The infectious disease trap of animal agriculture

**DOI:** 10.1126/sciadv.add6681

**Published:** 2022-11-02

**Authors:** Matthew N. Hayek

**Affiliations:** Department of Environmental Studies, New York University, 285 Mercer St., New York, NY 10012, USA. Email: matthew.hayek@nyu.edu

## Abstract

Infectious diseases originating from animals (zoonotic diseases) have emerged following deforestation from agriculture. Agriculture can reduce its land use through intensification, i.e., improving resource use efficiency. However, intensive management often confines animals and their wastes, which also fosters disease emergence. Therefore, rising demand for animal-sourced foods creates a “trap” of zoonotic disease risks: extensive land use on one hand or intensive animal management on the other. Not all intensification poses disease risks; some methods avoid confinement and improve animal health. However, these “win-win” improvements alone cannot satisfy rising meat demand, particularly for chicken and pork. Intensive poultry and pig production entails greater antibiotic use, confinement, and animal populations than beef production. Shifting from beef to chicken consumption mitigates climate emissions, but this common strategy neglects zoonotic disease risks. Preventing zoonotic diseases requires international coordination to reduce the high demand for animal-sourced foods, improve forest conservation governance, and selectively intensify the lowest-producing ruminant animal systems without confinement.

## Teaser

Animal agriculture creates a “trap” of rising infectious diseases, and escaping it requires limiting meat consumption.

## INTRODUCTION: FOOD PRODUCTION DRIVES ZOONOSIS EMERGENCE

Despite global advances in prosperity, nutrition, and medical care, infectious diseases are rising in prevalence (*1*, *2*). In the past four decades, emerging infectious diseases have increased at more than four times the rate of prior decades (*3*), most of which have nonhuman animal (zoonotic) origins.

Since 1940, an estimated 50% of zoonotic disease emergence has been associated with agriculture (*1*–*3*). This estimate, however, is necessarily conservative because only direct agricultural drivers are considered in the epidemiological literature, i.e., within the farm gate. Food systems have environmental impacts before and after the farm gate (*4*), such as land clearing, food processing, and waste disposal. Food systems therefore affect zoonotic disease emergence indirectly. The true contributions of food systems to recently emerged zoonotic diseases remain poorly characterized.

The increase in zoonosis emergence has been partially attributed to ongoing deforestation, particularly in the tropics (*2*, *5*, *6*). The largest driver of deforestation is pasture expansion for ruminants (e.g., cattle) with another substantial fraction of forest and savanna clearing for producing feed crops like soy, predominantly fed to monogastrics (e.g., pigs and chickens) for domestic and export markets (*7*), with ongoing debate as to the precise proportions (*8*). Land clearing is expected to continue through 2050 due to further increased meat and dairy demand (*9*–*12*). Deforestation and conversion to human-dominated systems drive the loss, turnover, and homogenization of biodiversity and expose adjacent human communities to wildlife harboring microbes that can become zoonotic pathogens with pandemic potential (*5*).

To meet the rising global demand for animal-sourced foods, the most commonly recommended development strategy in the environmental literature is “sustainable intensification,” which refers to increasing production while managing inputs more judiciously (*13*, *14*). Experts recommend this strategy for virtually all low- and middle-income countries (LMICs). By improving resource use efficiency, sustainable intensification strategies for animal agriculture can reduce greenhouse gas (GHG) emissions and deforestation (*15*–*17*), thereby also reducing zoonotic disease risks.

However, the intensification of animal agricultural production, in its most common forms, entails the concentration and confinement of animal bodies and their wastes, trading off deforestation for other multiple well-documented and potentially cascading risks for zoonotic disease emergence. This creates a paradox for intensification that remains unaddressed in the scientific literature: Intensified animal production, while decreasing marginal land use change and GHG emissions, can often increase other zoonotic disease risks. The risks of zoonotic disease emergence from intensive animal agriculture could therefore undermine the “sustainable” nature of sustainable intensification.

This review examines the zoonotic disease paradox inherent to the sustainable intensification of animal agriculture, exploring whether food systems can circumvent a “trap” of zoonotic disease risks as they further develop. The review first aims to characterize interactions between intensification and deforestation while examining ways that they both contribute to zoonotic disease risk. On the basis of these interactions, this review provides recommendations to reduce the likelihood of zoonotic disease emergence, including (i) selectively intensifying the least productive regions, namely, LMICs, without resorting to confinement and other common high-risk intensive management techniques; (ii) strengthening and improving conservation regulations with effective community governance; and (iii) curbing the high and rising demand for animal-sourced food products. These three strategies are most likely to succeed if implemented in tandem and via regional and international coordination to avoid leakage and rebound effects.

## INTENSIFICATION—RISKS, OPPORTUNITIES, AND LIMITS FOR STEMMING ZOONOTIC DISEASE

A number of intensive animal production methods have been implicated in zoonotic disease emergence in the literature (Table 1). The intensification of animal agriculture through confinement and industrialization has directly led to the emergence of viruses including Nipah and H5N1 influenza (“swine flu”) (*18*) and antibiotic-resistant infectious bacteria including methicillin-resistant *Staphylococcus aureus* and *Escherichia coli* (*19*, *20*).

**Table 1.** Intensive animal management strategies, by qualitative risk categories and farmed animal types.

**Elevated risks**	**Evidence of zoonotic disease emergence**
All farmed animal species	
Indoor production and confinement	(*83*–*85*)
Genetic homogenization	(*86*, *87*)
Subtherapeutic and growth- promoting antibiotic use	(*19*, *20*, *25*, *74*, *88*–*90*)
Long-distance transportation	(*91*, *92*)
Physiological stress from crowding, confinement, and conflicts (e.g., gestation crates, veal crates, and battery cages)	(*22*, *23*, *26*, *93*)
Temporary/seasonal and transient human labor	(*83*, *94*)
Concentrated animal wastes	(*88*, *95*)
**Neutral or reduced risks**	**Evidence of reduced land and resource needs**
All farmed animal species	
Improving veterinary care and reducing mortality	(*15*)
Improving animal husbandry management (e.g., lower reproductive age)	(*15*, *96*)
Integrating crop and livestock production	(*97*–*99*)
Ruminant species only	
Optimizing grazing densities	(*100*, *101*)
Improving forage quality	(*15*, *102*)
Amending and restoring degraded pastures	(*15*, *102*–*104*)

Intensified animal agriculture is often, but not always, characterized by a shift toward “landless” or “industrialized” systems (as defined by the United Nations Food and Agriculture Organization). These systems typically restrict animal movement and are oriented toward rapid weight gain and productivity (*21*). Monogastric animals like pigs and chickens are raised indoors in sheds, each animal with less than twice the space that their bodies occupy, with little or no room to express natural behaviors (*22*, *23*). Many beef cattle spend the latter part of their lives being “finished” or rapidly fattened to reach their final market weights on enriched feeds in feedlots, with stocking densities for cattle on outdoor feedlots of less than 4 m^2^ per steer/heifer (*24*). These environments entail physiological and mental stress, close proximity to each other and wastes, and the routine administration of subtherapeutic (infection-preventing) and growth-promoting antibiotics (Table 1). Zoonotic diseases from aquatic animals are relatively less common and are predominantly caused by bacteria rather than viruses (*25*). However, aquatic animal bacteria are expected to become more prominent and potentially infectious among humans as finfish aquaculture continues to grow to produce a larger share of aquatic foods globally, and with it are confinement, stress, and antibiotic use, potentially leading to spillover into humans (*26*). These intensive systems are predominant in developed, industrialized countries but are rapidly proliferating in developing regions (*27*), with encouragement and financing from international development organizations including the World Bank (*28*).

Relatively more extensive systems include pastoralism, extensive grazing, and mixed crop-livestock grazing. Extensive systems are used almost exclusively in developing regions, namely, through the tropics and semitropics, and among predominantly ruminant livestock (e.g., cows, buffalo, sheep, and goats).

Intensification methods sit on a spectrum, with poles of landless, industrialized production on the high end and highly extensive pastoralist grazing on the lowest. The most extensive and inefficient systems have the potential to be improved using “win-win” forms of intensification that do not entail a fully industrialized or landless kind of confined intensification (Table 1), but rather a kind of “meeting in the middle” for the lowest, least productive systems to improve their performance (*15*). Thus, intensifying low-production ruminant systems in a selective manner could confer a neutral or decreased risk of zoonosis emergence while improving meat and dairy productivity in the most marginal contexts.

However, there are limitations to this form of intensification. First, the number of animals raised in extensive systems is already decreasing while being supplanted by highly industrialized/landless systems throughout developing regions (*11*, *21*). Therefore, there are regional and global limitations to how much additional food “semi-intensive” systems can provide. Second, shifts downward from more highly intensive forms would compromise food production or lead to net agricultural expansion. For instance, eliminating feedlot beef cattle systems in the United States by shifting to intensive grazing would require 64 to 270% greater land use (*29*), while eliminating confined indoor broiler chicken systems by shifting to minimal pasture would require 43.8 to 60.1% greater land use (*30*). Industrialized systems are often more productive and resource efficient than semi-intensive methods. Shifting away from industrialized systems therefore entails a GHG and land use penalty or “sustainability gap” (*30*). Last, production systems for monogastric animals, which produce two-thirds of meat globally, lack common semi-intensive commercial methods (*21*). Global production and consumption of beef, pork, and chicken are expected to rise by 39, 55, and 58%, respectively, by 2050, with the majority of additional production expected to be achieved through intensification systems (industrial, in the case of monogastrics) (*11*). Therefore, additional food system strategies beyond intensification are needed to safely feed a rising and more affluent global population.

## INTERACTIONS BETWEEN INTENSIFICATION AND DEFORESTATION

### Intensification tends to reduce deforestation directly

**Fig. 1.**
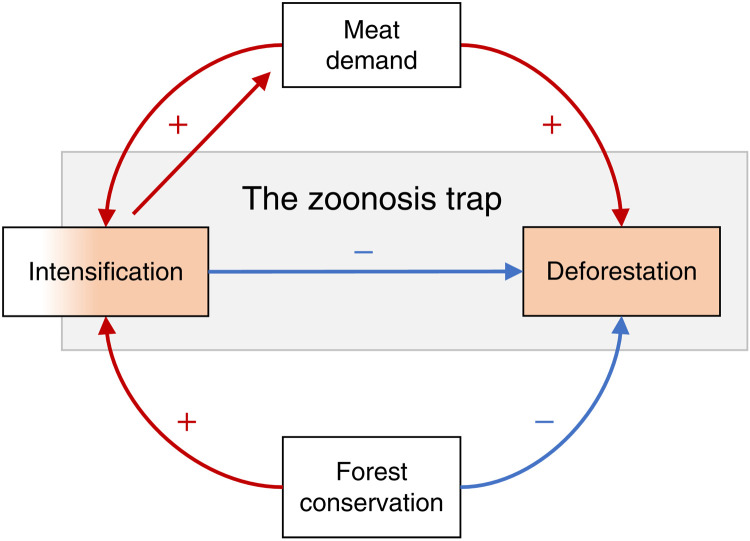
Higher incomes are associated with high meat demand that must be met through intensification or deforestation (or both). Intensification can trigger higher meat demand through lower prices, because meat demand is elastic with respect to its cost. Intensification and deforestation are highlighted in orange, as both have caused recent zoonotic disease emergence and are predicted to continue doing so. Intensification is colored by a gradient to indicate that intensification strategies lie on a gradient of helpful/neutral to harmful with respect to zoonosis risks.

Intensification, which aims to make agricultural production more efficient, is commonly understood to decrease the pressure for deforestation within the environmental literature (*13*, *31*, *32*). However, in many developing tropical regions, both intensification and deforestation are occurring simultaneously because they share underlying drivers (i.e., confounding causes): rising populations, incomes, and demand for animal-sourced foods (Fig. 1). Because the two are visibly correlated, the epidemiological literature on zoonotic disease often erroneously links intensification directly to deforestation. A number of recent high-profile synthesis reports on zoonoses discuss intensification and deforestation synonymously and interchangeably (*6*, *33*, *34*), sometimes directly implicating intensification as causing the ongoing deforestation, although the environmental literature predominantly concludes the opposite. Intensification can lead directly to reduced deforestation in agriculture-forest frontiers (*35*, *36*).

### Intensification can indirectly trigger more deforestation

Intensification reduces the marginal resource requirements of animal-sourced food production; it thus can potentially reduce pressures for deforestation, a finding that is widely accepted and uncontroversial. However, after achieving higher efficiency, intensification can lower the costs of production and sale prices of final goods, inducing higher demand and production (Fig. 1). This greater demand can then incentivize additional deforestation (*37*), negating some or all of the original efficiency improvements. This trade-off is known as Jevons’ paradox (*36*, *38*, *39*) or “rebound effects,” more commonly.

The occurrence and magnitude of rebound effects in animal-sourced food production are difficult and controversial to identify because of confounding factors (*40*–*42*), leading to ongoing debates (similarly reflected in the “land sparing versus land sharing” debate regarding agricultural efficiency). However, some trends and investigations are illuminating. In Sweden and the United States, an increased consumption of chicken over the past two decades, due to lower prices, resulted in greater aggregate GHG emissions despite marginal efficiency gains over the same period (*43*, *44*). In South America, beef intensification has triggered further deforestation due to lower production costs (*35*, *37*, *45*). Sustainable intensification can thus spur greater environmental impacts, undermining its sustainable aims (*46*, *47*). Intensification is necessary but insufficient to reduce pressures for agriculture expansion and land clearing. Escaping this “damned if we do, damned if we don’t” trap of intensification (Fig. 1) requires a more multipronged approach.

### Effective forest conservation occurs in tandem with other strategies

Intensification alone is an insufficient strategy for reducing zoonotic disease risk (see the “Intensification—Risks, opportunities, and limits for stemming zoonotic disease” section) and for mitigating and reversing deforestation (see the previous section). Direct forest conservation policies and incentives are widely recommended in environmental and epidemiological literature, e.g., (*6*, *18*, *33*). However, known trade-offs and pratfalls exist. First, forest and wildlife habitat conservation policies that are not appropriately designed and enforced with the involvement of local cultures have backfired (*36*, *48*–*50*). Second, conservation may lead to “leakage” effects: Globalization allows production to relocate, along with its deforestation, to countries where conservation policies are insufficiently adopted or enforced (*51*, *52*). Last, effective forest conservation policies in the short term can boost intensification but lead to further deforestation in the long term and across wider regions (Fig. 1) (*39*). These effects can vary over space and time, changing with local livelihoods and culture, price elasticities for agricultural goods, and how connected production regions are to global markets (*37*).

Conservation policies should be culturally sensitive, rigorously enforced, and have long-term community buy-in. However, a well-crafted conservation policy is still insufficient to spare land from agricultural pressures; additional land for rising populations and diets richer in animal-sourced foods must come at the expense of clearing native habitats somewhere (*11*, *53*).

## MEAT DEMAND AT THE NEXUS OF ENVIRONMENTAL CHANGES

The largest increases in meat demand and production are occurring in developing, tropical regions (*16*). Meat consumption exceeds the dietary requirements in high-income countries and among increasingly urban and middle-class populations of most middle-income countries (*54*–*56*). As demand rises along with affluence in the coming decades in LMICs and high-income countries continue to sustain high levels of consumption and exports, additional land clearing and GHG emissions will occur even with ambitious levels of intensification (*9*, *12*).

### Shifting to plant-rich diets mitigates environmental and zoonotic disease risks

Decreasing meat consumption has cobenefits for environmental protection and zoonotic disease risks. Global dietary changes are theoretically sufficient to reverse ongoing deforestation trends, providing 5 to 11 GtCO_2_ per year of natural carbon removal across 5 to 12 million km^2^, sequestering approximately a decade worth of anthropogenic emissions by 2050 in natural vegetation (*9*, *57*–*59*), which would also conserve and restore a substantial fraction of lost biodiversity (*53*, *60*). Shifts to plant-rich diets in high-income countries alone would remove approximately 3 million km^2^ from agricultural production, including 1 million km^2^ of natively forested areas (*9*, *56*).

**Fig. 2.**
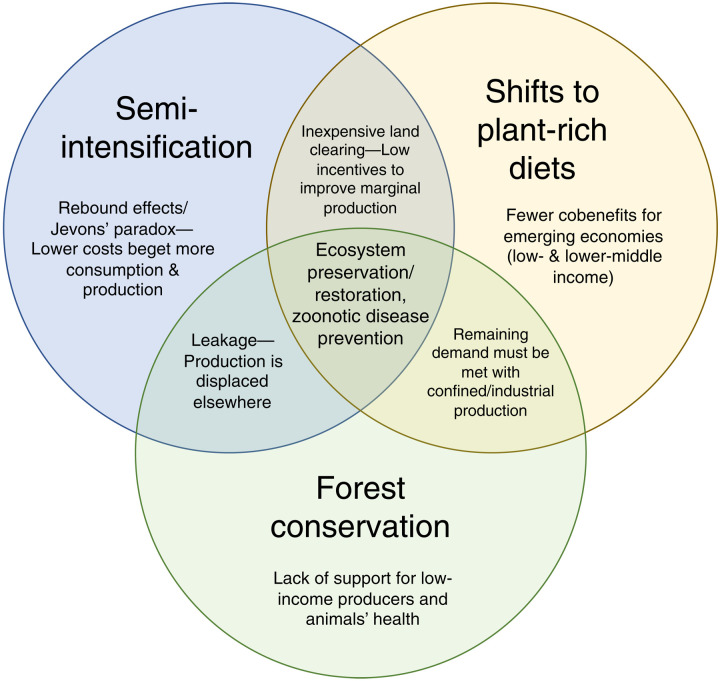
A three-pillar approach for preventing zoonotic disease emergence and reducing environmental impacts from animal agriculture (center). Within individual circles and the intersections between the two, limitations of adopting only one or two strategies are described.

To address the emerging zoonotic disease risks of animal agriculture, a multipillared approach is required (Fig. 2). This approach includes reducing demand for animal-sourced foods, semi-intensification (see the “Intensification—Risks, opportunities, and limits for stemming zoonotic disease” section and Table 1), and direct forest conservation (see the “Effective forest conservation occurs in tandem with other strategies” section). Under business-as-usual conditions of rising demand for animal-sourced food, increased land clearing is inevitable (*57*, *61*). Reducing demand can therefore avoid leakage and rebound effects from focusing exclusively on supply-side protections like semi-intensification and forest conservation (Figs. 1 and 2).

The zoonotic disease risks of rising animal-sourced food production and consumption have been underscored by a number of recent major environmental epidemiology synthesis reports (*6*, *33*, *62*). These reports imply or outright state that high future demand for animal-sourced foods is an immutable consequence of rising incomes, treating this trend as fait accompli rather than a decision point for policy interventions. This fatalism contradicts behavioral science research on reducing the consumption of meat and other products with harmful public health impacts (e.g., tobacco and sugar).

To meaningfully flatten the rising curve of animal-sourced foods, demand-side interventions should be implemented, tested, and scaled ambitiously (*63*). Even gentle changes to dining options and presentation can create large effects (*64*). Effective interventions range from these subtle “nudges” to more blatant rewards and incentives, as well as stringent regulations and restrictions (*16*, *55*). This spectrum has been described using the Nuffield intervention ladder, with lower rungs of “soft” methods or “carrots” (e.g., guidance, suggestions, education, and nudging) to higher rungs of increasingly forceful “hard” interventions or “sticks” at the top (e.g., taxes and bans) (*65*).

Countries lack healthy and sustainable food consumption policies that are comprehensive and synergistic; most countries only have education policies (e.g., dietary recommendations), with higher rungs on the Nuffield ladder—including guiding choices through changing incentives and defaults or disincentivizing options—completely missing (*66*). Promising local policies and corporate initiatives, meanwhile, are aiming to guide consumers toward more sustainable options using methods of monitoring, goal setting, and verification in combination with multiple soft behavioral interventions to motivate change (*67*).

More targeted dietary change interventions are needed; recommendations for dietary change policies across most scientific literature are general and vague (*16*, *55*). Policies can leverage social, behavioral, and organizational sciences to change the underlying motivations and choice environments that drive consumer decisions (*64*, *67*). Small successes should also be better communicated to decision-makers and ambitiously scaled to large populations with help from community-based advocacy and organizing (*68*).

### Differentiating risks across food animals

Shifting production and consumption from beef to poultry is a common recommendation in the literature. Such shifts would accomplish most of the GHG emission mitigation as reducing or eliminating all meat (*69*–*71*). These recommendations have shaped national climate policies: Ethiopia stated plans to shift 30% of their beef production to poultry in their 2021 Nationally Determined Contribution to the United Nations Framework Convention on Climate Change (*72*). However, such shifts could maintain or even increase zoonotic disease risks.

**Fig. 3.**
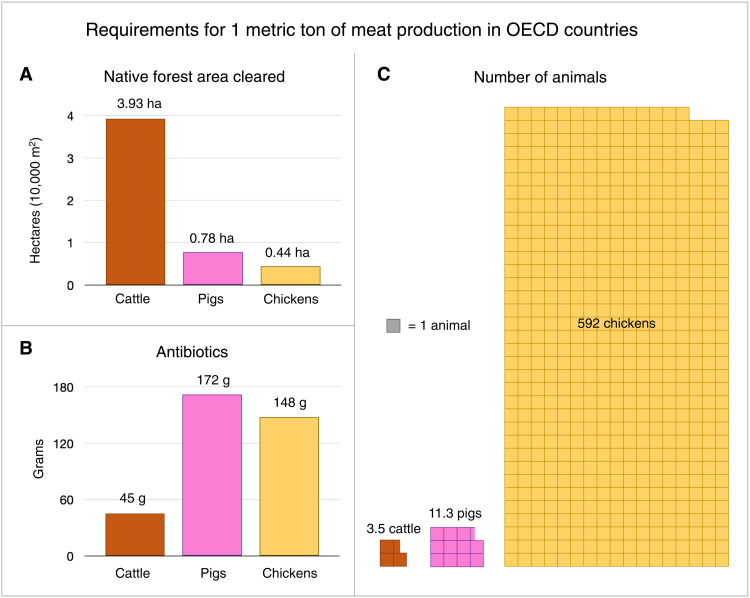
Requirements to produce 1 metric ton of meat (dressed carcass weight), averaged among all OECD countries and weighted by production quantity, base year 2010. (**A**) Hectares required for the production of animal feed (crops, pastures, and forages) in natively forested areas, calculated by the author from geospatial potential vegetation data and agricultural production data in (*9*) and sources therein. (**B**) Grams of antibiotics used, derived from (*74*). (**C**) Number of animals required for slaughter, from United Nations FAOSTAT (*105*). OECD, Organization for Economic Co-operation and Development.

Beef has higher land use and is associated with more tropical deforestation than any other commodity (*73*). However, monogastric animals, including pigs and chickens, require higher antibiotic use and higher animal populations to produce the same quantity of meat as ruminants such as cattle (Fig. 3). Pigs and chickens are fed more than three times the antibiotics than cattle in intensive systems (*74*) due to close confinement of animals and their wastes. It takes three pigs or 170 chickens to produce the meat of one steer. Intensive methods of monogastric animal production entail more marked confinement, including hen laying and pig gestation systems wherein animals are confined without enough space to spread their wings or turn around. Now, there are more than 33 billion chickens on Earth, representing more than 70% of global avian biomass (*75*). Shifts from beef to even greater chicken consumption would entail greater confinement and subtherapeutic antibiotic use for a larger number of animals, elevating multiple risks for zoonotic disease emergence.

The precise zoonotic disease risks of individual foods and whole dietary patterns have not previously been quantified. Statistical analyses are challenging because any predictive metrics would entail creating robust models from only a few (but highly costly) zoonotic disease spillover events and outbreaks that have emerged from agricultural production, often from diverse pathogens and with sometimes ambiguous origins. The lack of quantitative disease analyses remains a hurdle to assessing the full costs, benefits, and trade-offs of food system transitions. Despite this, plant-rich diets entail cost-saving cobenefits (*76*, *77*), including environmental outcomes, human nutrition, and animal welfare, which have been quantified robustly in previous work (*78*–*80*).

## INTERNATIONAL COORDINATION FOR PRIMARY PREVENTION OF PANDEMICS

The coronavirus disease 2019 pandemic has increased the vigilance of the global community in identifying and monitoring the potential sources of the next zoonotic disease outbreak. Well-trodden prevention strategies include suppressing disease in vulnerable animals, monitoring transmission and spillover events of pathogens with pandemic potential, and stopping detected outbreaks in domesticated animals through culling (*81*). These decade-long pursuits have only tackled pathogens of concern after some initial emergence or spillover. They do not address root causes of transmission, mutation, spillover, and proliferation of emerging infectious zoonotic pathogens. The high and increasing demand for animal-sourced foods is one such root cause.

Strategies that prevent infectious diseases at their root sources are called primary prevention (*6*, *18*, *33*). This work outlines three pillars for primary prevention that, when combined, constitute stronger protection against zoonotic diseases from animal agriculture than any one pillar in isolation (Fig 2). National governments should coordinate their support for a wide range of policies and activities that support these pillars, including expanding veterinary and extension services for improved animal care in LMICs (*18*), phasing out and banning subtherapeutic and growth-promoting antibiotic uses (*82*), forming multilateral commitments among countries importing and exporting tropical commodities linked to deforestation (*73*), ambitiously scaling community-based approaches to popularizing plant-rich diets (*68*), supporting open and public alternative protein research (*77*), and facilitating sustainable and just transitions for producers. Commitments should also set quantifiable science-based goals and fund ongoing research to monitor and accelerate progress. Together, the three pillars of primary prevention can guide and empower decision-makers to escape the zoonotic disease trap of business-as-usual animal agriculture.
